# The Use of miRNAs in Predicting Response to Neoadjuvant Therapy in Oesophageal Cancer

**DOI:** 10.3390/cancers14051171

**Published:** 2022-02-24

**Authors:** Cameron C. J. Lang, Megan Lloyd, Said Alyacoubi, Saqib Rahman, Oliver Pickering, Tim Underwood, Stella P. Breininger

**Affiliations:** Cancer Research UK Center, Faculty of Medicine, School of Cancer Science, University of Southampton, Southampton General Hospital, Southampton SO16 6YD, UK; cl8g17@soton.ac.uk (C.C.J.L.); meganlloyd@hotmail.co.uk (M.L.); said.alyacoubi@nhs.net (S.A.); s.rahman@soton.ac.uk (S.R.); o.j.pickering@soton.ac.uk (O.P.); t.j.underwood@soton.ac.uk (T.U.)

**Keywords:** oesophageal adenocarcinoma, oesophageal squamous cell carcinoma, predicting response, chemotherapy, chemoradiotherapy, neoadjuvant therapy, microRNAs

## Abstract

**Simple Summary:**

Chemotherapy and chemoradiotherapy are only effective in 25% to 30% of patients with oesophageal cancer. Being able to predict which patients will respond to chemotherapy and chemoradiotherapy before they receive this treatment will prevent patients undergoing unnecessary procedures that may reduce their quality of life and help source alternative treatment options faster. The scope of this review was to understand whether microRNAs, small non-coding RNA molecules that regulate gene expression, can be used as biomarkers to predict a patient’s response to chemotherapy and/or chemoradiotherapy treatment. This review showed that a number of microRNAs may have the potential to predict response to chemotherapy and chemoradiotherapy alongside other pre-treatment features already used. More research is needed to translate the use of microRNAs as biomarkers of response to the clinical setting, as well as understanding the effects different types of treatment have on predictability.

**Abstract:**

Oesophageal cancer (OC) is the ninth most common cancer worldwide. Patients receive neoadjuvant therapy (NAT) as standard of care, but less than 20% of patients with oesophageal adenocarcinoma (OAC) or a third of oesophageal squamous cell carcinoma (OSCC) patients, obtain a clinically meaningful response. Developing a method of determining a patient’s response to NAT before treatment will allow rational treatment decisions to be made, thus improving patient outcome and quality of life. (1) Background: To determine the use and accuracy of microRNAs as biomarkers of response to NAT in patients with OAC or OSCC. (2) Methods: MEDLINE, EMBASE, Web of Science and the Cochrane library were searched to identify studies investigating microRNAs in treatment naïve biopsies to predict response to NAT in OC patients. (3) Results: A panel of 20 microRNAs were identified as predictors of good or poor response to NAT, from 15 studies. Specifically, miR-99b, miR-451 and miR-505 showed the strongest ability to predict response in OAC patients along with miR-193b in OSCC patients. (4) Conclusions: MicroRNAs are valuable biomarkers of response to NAT in OC. Research is needed to understand the effects different types of chemotherapy and chemoradiotherapy have on the predictive value of microRNAs; studies also require greater standardization in how response is defined.

## 1. Introduction

### 1.1. Oesophageal Cancer Epidemiology

Oesophageal cancer (OC) is the ninth most common cancer diagnosed globally, yet the sixth most common cause of cancer related death [1], resulting in an estimated 436,000 patient deaths in 2017 [2,3]. Five-year survival for OC is 17% [4]. The prognosis is bleak when compared to other cancers such as colorectal cancer, of which the 5-year survival is almost 3 times greater than that of OC [5]. These poor outcomes can at least in part be attributed to late presentation, with 47.9% of patients diagnosed at stage 4 [6]. OC is more common in men who are 3 to 4 times more likely than women to develop OC, and the median age of diagnosis is 68 years [1]. There are two major OC subtypes, oesophageal squamous cell carcinoma (OSCC) and oesophageal adenocarcinoma (OAC), for which OSCC estimated to contribute for over 70% of all OC diagnoses globally [7,8]. OSCC, shows greater prevalence across Asia within an “oesophageal cancer belt” that stretches from north-east Iran to north-west China [9]. This is likely due to the higher prevalence of tobacco use, along with genetic differences in alcohol metabolism which leads to acetaldehyde accumulation, a known carcinogen [7,10]. OAC is, however, predominant in the Western world, probably due to the high incidence of obesity and gastro-oesophageal reflux disease [11] which are the primary risk factors for OAC development. The UK has the highest incidence of OC in Europe with approximately 10,000 new cases per year [12]. The UK’s 5-year survival for OC is 12% in line with average across Europe; however this varies largely from 9% in Latvia to 25% in Belgium [4].

### 1.2. Pathophysiology of OAC and OSCC

OAC is most often found in the lower part of the oesophagus and at the gastro-oesophageal junction, where it frequently develops from its precursor Barrett’s oesophagus [13]. Persistent exposure to acid and bile reflux, results in mucus-secreting glandular metaplasia [14]. Increased genetic mutations and loss of heterozygosity are seen during epithelial proliferation. Notably in the progression to OAC two defining genetic mutations are present that can be utilised to differentiate between non-dysplastic Barrett’s oesophagus, high grade dysplasia and OAC tissues, these are *TP53* and *SMAD4*. Mutations in the tumour suppressor gene *TP53* occur in 50–71% of all OAC cases [7] but have been shown to be recurrently mutated in high grade dysplasia (HGD) and OAC samples. *SMAD4* mutation is shown to be exclusive to OAC tissue and thus it can be concluded it lies at the boundary between progression from HGD to OAC [15]. The loss of these tumour suppressor genes (TSG) or mutation of proto-oncogenes leads to dysplastic Barrett’s due to a greatly increased rate of uncontrolled cellular proliferation [7]. Further mutagenic changes and chromosomal instability over time result in the formation of OAC and without intervention, infiltration of the basement membrane and subsequent metastasis.

OSCC pathology is subtly different; it more commonly presents in the middle and upper parts of the oesophagus [16]. Persistent physical insults, primarily chronic alcohol and tobacco use, lead to squamous cell hyperplasia [17]. Over time should exposure be unchanged, genetic mutations will accumulate due to greatly increased rates of cellular proliferation. *TP53* mutation in OSCC is almost universally found in all patients, approximately 92% [7,18]. However, *NOTCH1* and *NOTCH3* mutations have been shown to be significantly more frequent in OSCC than OAC, at 33% versus 25%, respectively [18]. These mutagenic accumulations eventually result in a loss of negative feedback mechanism due to malfunction of tumour suppressors. The result is uncontrolled rapid rates of cellular proliferation producing a carcinoma [7].

### 1.3. Current Pathways of Screening

Currently there is no national screening programme for OC in the UK [19], in contrast to Asian countries such as Japan and Korea where a higher disease incidence is seen. These have been shown to significantly improve outcomes [20,21], likely due to earlier detection and thus earlier intervention. Screening programmes consist of fibreoptic upper gastrointestinal endoscopy or Barium Upper Gastrointestinal Series for those over 40 years, recommended every 2 years thereafter [20,21]. With these interventions approximately two thirds of upper GI cancers are detected at an early stage, compared to the 70% of late-stage diagnoses across Europe [1,20,21]. The largest issue with screening for OC is the invasiveness of this procedure and therefore the risk it poses to the patients [19], particularly should these results be negative and therefore futile. Mass screening would therefore place 1 in 200 to 1 in 10,000 patients at risk of adverse events such as infection, perforation and bleeding [22]. The American and British Societies of Gastroenterology suggest screening for OAC should be provided to patients with reflux > 5 years, Caucasian males and family history of Barrett’s oesophagus or OAC [7]. Despite recent evidence showing one off endoscopic screening in China led to a reduction in incidence and mortality, no current OSCC screening is in place [23] possibly due to its lower population prevalence in the UK. Traditionally, there have been no minimally invasive procedures with high enough sensitivities to consider their use in widespread screening programmes. However, new research into Cytosponge™ technology for detection of Barrett’s oesophagus and early dysplasia suggests a specificity and sensitivity of 79.9% and 92.4% respectively, which is comparable to current screening programmes for colorectal cancer in the UK with a false positive rate of 2–9% [24], as well as improved detection rates of Barrett’s oesophagus in the primary care setting [25]. Utilisation of this minimally invasive sampling method, alongside modern genetic testing could prove to be a highly sensitive and specific way of detecting and tailoring treatment regimens to patients.

### 1.4. Treatment of Oesophageal Cancer

**Figure 1:**
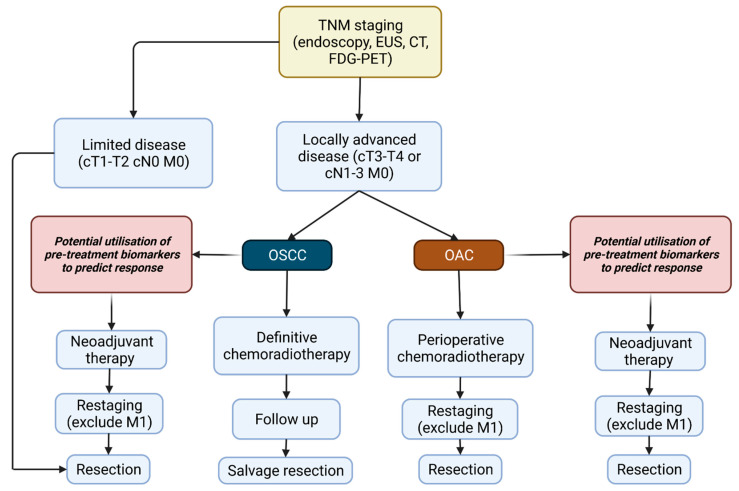
Current treatment strategies for oesophageal cancer as outlined by the European Society of Medical Oncology. TNM staging: T describes tumour size and any cancer spread into adjacent tissue; N describes cancer spread to adjacent lymph nodes; M describes metastasis (Adapted from “Oesophageal Cancer: ESMO clinical Practice Guidelines” (2016) [27]. Created with BioRender.com, accessed on 17 February 2022).

Where OC is potentially curable at presentation, it is locally advanced in the majority of cases. Standard of care treatments in this setting for both OSCC and OAC are usually extensive and invasive, requiring neoadjuvant chemotherapy or chemo-radiotherapy (NAT) followed by surgical resection as recommended by NICE guidelines [26]. Guidance on the treatment pathway of OSCC and OAC based upon staging and functional assessment of the patient, as recommended by the European Society of Medical Oncology are outlined in Figure 1 [27]. A variety of studies have investigated the benefits of NAT. The MRC MAGIC trial showed patients who receive a neoadjuvant regimen of epirubicin, cisplatin and 5-fluorouracil (5-FU) (ECF) therapy had a higher rate of overall survival (5-year survival, 36% vs. 23%) and progression free survival (0.53 to 0.81, *p* < 0.001) in comparison to patients undergoing surgery alone [28]. Similarly, the CROSS trial demonstrated that neoadjuvant chemoradiotherapy improved median overall survival from 24 to 49.4 months vs. surgery alone [29]. However, only 25% to 30% of patients achieve a partial or complete pathological response [30,31], and it carries a 0.5 to 2% mortality rate [32]. Early identification of patients that respond well could improve outcomes by preventing the administration of treatment regimens that are unlikely to be effective and facilitating treatment modulation [33]. Response to therapy is usually assessed via assignment of the Mandard Tumour Regression Grade (TRG) ranging 1 to 5 [34]. Responders are usually defined as TRG 1 (complete regression with no viable tumour cells evident) and TRG 2 (presence of residual cancer cells), at least for patients receiving chemotherapy. By administering NAT in patients who do not respond well, surgery is delayed, which if carried out earlier may have proven more effective. The main benefits of NAT are the increased chance of complete resectability of the primary tumour, as reduced tumour mass induced by NAT decreases the area of resection required, as well as improved prognostic outcome due to the decreased incidence of nodal micrometasteses [32,35,36]. On the contrary, tumour progression during therapy can occur in those patients who do not respond well to NAT or conversely overtreatment of tumours with a favourable prognosis that are unlikely to respond to NAT. Therefore, identifying biomarkers that allow successful identification of who will or will not respond to therapy are desperately needed to allow rational treatment decisions to be made.

### 1.5. Function of miRNAs and Their Role in Cancer

**Figure 2:**
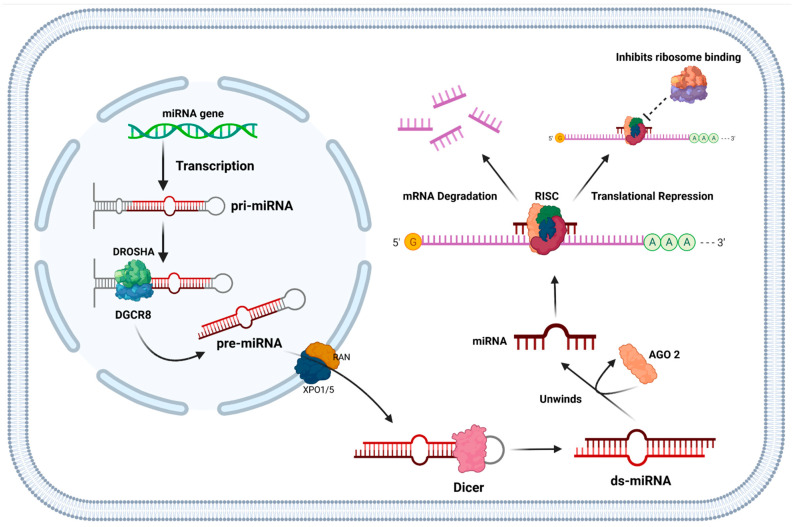
Synthesis and action of miRNA in post-transcriptional gene regulation. Transcription of miRNA gene via RNA Polymerase II forms pri-miRNA, DROSHA (class 2 ribonuclease III enzyme) and DGCR8 cleave the terminal end of the miRNA hairpin to form pre-miRNA. This is exported via RAN and XPO1/5. The miRNA hairpin is then cleaved by Dicer. AGO-2 binds to the double stranded miRNA, unwinds and dissociates the strands then forms a complex with RISC. This leads to either miRNA degradation or inhibition of ribosome binding. Abbreviations: pri-miRNA: primary-miRNA, pre-miRNA: precursor-miRNA, DGCR8: DiGeorge syndrome critical region gene 8, XPO 1/5: Exportin 1/5, RISC: RNA-induced silencing complex. (Created with BioRender.com, accessed on 16 January2022).

MicroRNAs (miRNAs) are single stranded noncoding RNA molecules approximately 22 nucleotides long, regulating gene expression at the transcriptional or post-transcriptional level [36,37]. They do this by binding in a specific sequence to the complementary region in the 3′ untranslated mRNA region, which then regulates the translation of mRNAs to proteins [38]. miRNAs can bind to a complementary mRNA region resulting either in blockage of translation or degradation of a section of miRNAs via the RNA-induced Silencing Complex (RISC) complex, both leading to inactivation of a gene (Figure 2) [37,39]. Common molecules regulated are signalling proteins as well as transcription factors to RNA binding proteins [40]. miRNAs play an important role in biological pathways and their expression is dysregulated in multiple pathological mechanisms [41]. Aberrant miRNAs expression patterns are involved in the initiation and progression of oncogenesis due to their role as TSG and oncogenes. Oncogenic miRNAs target and prevent the expression of endogenous TSG, which activate pathways associated with OC, such as the reduced expression of miR-27a leading to permanent activation of the *KRAS* pathway [42]. TSG are often downregulated, dysfunctional or completely lost in OC such as miR-30b-5p [43] or miR-34a [44] in OSCC whereas those associated with proto-oncogenes are upregulated [45].

Recent research into the effects of NAT on miRNAs expression in other cancers such as breast cancer and rectal cancer have shown promising results. Over the course of NAT Lindholm et al. (2019) [46] showed that tumour suppressor miRNAs expression, such as miR-100-5p and miR-125b, were upregulated following treatment [46] which may reflect a role in the regulation of chemosensitivity. Kheirelseid et al. (2013) [47] identified expression signatures of miR-16, miR-590-3p and miR-561 that were predictive of complete versus incomplete response to neoadjuvant chemoradiotherapy in pre-treatment samples of rectal cancer [47]. A recent study showed that several miRNAs can predict poorer overall survival in both OSCC and OAC [48] such as the upregulation of miR-21 and downregulation of miR-133a. Hence, using miRNAs as predictors of pre-treatment response as well as other factors such as survival, seems to be a viable non-invasive potential solution to improving the accuracy of patient allocation to treatment.

Currently, there is no biomarker for stratifying patients into responders and non-responders to NAT using treatment naïve samples. Utilising circulating miRNAs to predict response to NAT could provide a simple and minimally invasive solution such as blood sampling could be used to identify patients that will benefit from NAT, thus improving outcome and quality of life. This could be utilized to facilitate optimal treatment choices and the likelihood of patients achieving a pathological good or complete response. Here we review 15 studies that investigated whether miRNAs could be used as a novel biomarker for predicting the response to neoadjuvant therapy, via the use of pre-treatment sample analysis in patients with OAC or OSCC.

## 2. Materials and Methods

**Figure 3:**
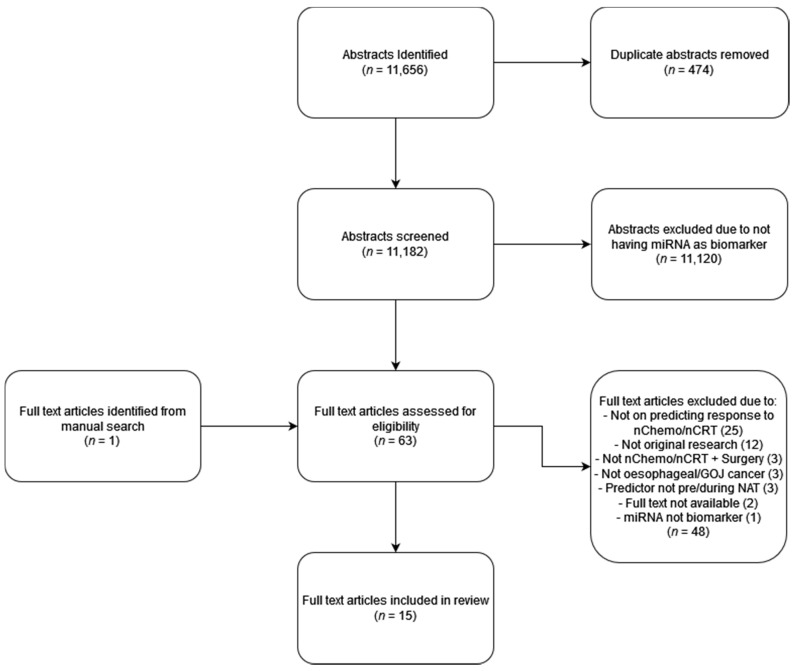
Schematic illustration of review articles included in this manuscript.

Online databases including MEDLINE, EMBASE, Web of Science and Cochrane library were searched to identify relevant studies investigating the use of miRNAs to predict response to NAT in OAC and OSCC (Figure 3). Articles were selected based on the following criteria. All participants must be ≥18 years diagnosed with locally advanced OAC or OSCC; treatment via neoadjuvant chemotherapy or chemoradiotherapy followed by surgical resection; histological samples were pre-treatment samples, the rationale being that assessing post-treatment samples would not “predict” response but simply evaluate a response that has already occurred. Standard dataset restrictions were placed upon each database, these being English language only and from 1 January 1990 onward. The search strategy devised was modified as per each database syntax requirements with MeSH terms being utilised for MEDLINE and Cochrane library databases. The main concepts included in this search were oesophagus/gastro-oesophageal junction, cancer, response, chemotherapy/chemoradiotherapy and miRNA. Boolean AND/OR operators along with truncation and wildcard syntax were then used to link and expand search terms.

## 3. Results

### 3.1. MiRNAs Used to Identify Response in OAC Patients

Out of the 15 studies identified five studies looked exclusively at patients with OAC histology. Three studies utilised frozen OAC samples [49,50,51], one used Formalin-Fixed, Paraffin-Embedded (FFPE) samples [52] and the last study did not define the environment histological samples were stored under [45]. Four articles studied patients treated with neoadjuvant chemotherapy only and one studied neoadjuvant chemoradiotherapy patients. Table 1 describes the key results from the 5 identified studies in OAC, which will be described in detail in this section.

#### 3.1.1. Articles Studying Response to nChemo Only in OAC Patients

Bibby et al. (2015) utilised pre-treatment OAC biopsy specimens (*n* = 18) to screen for miRNAs by miRNA profiling arrays [49]. MiRNA expression was then assessed via quantitative polymerase chain reaction (qPCR). Patients were administered Cisplatin and 5-Fluorouracil followed by 1.87 Gy/min radiation prior to surgery. Out of 742 miRNAs expressed intratumorally, 67 were differentially expressed between responders and non-responders. MiR-330-5p expression was most differentially expressed between responders (*n* = 9) and non-responders (*n* = 10), showing significantly elevated expression levels in pre-treatment samples of responders (*p* < 0.01). Utilisation of clonogenic survival assay to assess alterations of cell line (OE19 and OE33) sensitivity to chemoradiotherapy showed a statistically significant increase in radio-resistance of cell lines with miR-330-5p silencing. Thus, it concluded that miR-330-5p downregulation may act as a potential biomarker for predicting complete pathological response in patients receiving neoadjuvant chemoradiotherapy.

Lynam-Lennon et al. (2016) analysed pre-treatment frozen biopsies from 18 patients receiving Cisplatin and 5-Fluorouracil followed by 40.05 Gy total radiation dose in 15 daily fractions [50]. A TaqMan miRNA assay was used to analyse the abundance of 742 miRNAs, of which 67 were differentially expressed between responders (*n* = 8) and non-responders (*n* = 10). Of these samples, expression of 35 miRNAs were increased in non-responders, classified as TRG4 and TRG5. Of particular note is miR-187, which was expressed in every tumour sample; it was significantly lower (*p* = 0.005) in patients classified as non-responders when compared with responders. Another similar study by Lynam-Lennon et al. (2012) in frozen OAC samples (*n* = 19) showed miR-31 to be significantly higher in ‘good’ responders (*n* = 9) defined as patients with TRG 1-2, when compared to ‘poor’ responders (*n* = 10) (TRG 4–5) [51].

Finally, Skinner et al. (2014) carried out a 3-step study across 3 patient cohorts (total *n* = 118) treated with Cistplatin and 5-Fluorouracil [45]. The discovery cohort (*n* = 10) were used to identify miRNAs present in resected specimens. A total of 754 miRNAs were examined in pre-treatment biopsies of 10 patients in the discovery cohort via TaqMan array. Median expression of each of these miRNAs in tumour samples was determined and compared between complete responders (*n* = 5) and non-complete responders (*n* = 5). Forty-four miRNAs most able to discriminate between these groups entered the model cohort (*n* = 43) for further analysis via a Fluidigm array. Of these 44 miRNAs, 4 (were miR-505; miR-99b; miR-451 and miR-145) were found to be significantly differentially expressed (*p* = 0.008) between pathological complete response (pCR) and non-pCR, showing elevated expression levels in non-responders. A logistic regression classifier combining these 4 miRNAs (termed the miRNAs Expression Profile (MEP)) and clinical variables was derived and then validated on a separate cohort of 65 patients. This classifier achieved good accuracy in discriminating between patients achieving a pCR and those who did not, with an area under the receiver operator characteristic curve (AUROC) of 0.77 seen.

#### 3.1.2. Article Studying Response to nCRT in OAC Patients

Ilhan-Mutlu et al. (2015) assessed response to neoadjuvant chemotherapy (nChemo) regimen only with Docetaxel (*n* = 8); Mitomycin/Cisplatin/5-FU (*n* = 8); Cisplatin/5-FU (*n* = 10); Epirubicin/Oxaliplatin/Capecitabine (*n* = 1) and Others non-specified (*n* = 6), in 36 OAC patients by quantifying miR-21 and miR-148a levels in pre-treatment surgical specimens [52]. Pathological complete response of patients was later defined using the Mandard Tumour Regression Grade, TRG 1 (complete regression), and no significant difference in expression levels of miR-21 and miR-148a were noted between responders (*n* = 1) and non-responders (*n* = 35). Thus, they concluded miR-21 and miR-148a were not predictors of response to NAT in OAC. However, due to the small sample of only one responder to compare the miRNAs no conclusions can be drawn.

### 3.2. MiRNAs Used to Identify Response in OSCC Patients

Of the 15 studies identified utilising OSCC samples exclusively (Table 2), one study used frozen and FFPE OSCC tissue samples [53] while the rest investigated circulating miRNAs in OSCC patients [54,55,56,57,58,59]. Five articles studied patients exclusively treated with nCRT and the other three studied patients treated with nChemo only.

#### 3.2.1. Articles Studying Response to nCRT in OSCC Patients

Han et al. (2019) examined pre-treatment serum samples of 104 OSCC patients and found that high miR-338-5p expression predicted pathological complete response. Low histopathological response was classified as grade 1a (more than two thirds residual tumour cells) or grade 0 (no significant response to chemotherapy) according to the guidelines of the Japan Esophageal Society (JES) [55,56,60]. Pre-treatment serum miR-330-5p concentrations were inversely correlated with post-therapy pathologic ypT-stage (*p =* 0.034), ypM (*p =* 0.014) and overall pathological ypTNM stage (*p =* 0.017). Chan et al. (2017) [54], Niwa et al. (2019) [58] and Wen et al. (2016) [53] produced results related to variants of miR-193, these were miR-193b, miR-193b-5p and miR-193b-3p. There structures are identical yet originate from different arms of the same pre-miRNA, with one usually being in higher cellular abundance than the others [61]. Chan et al. (2017) prepared pre-treatment serum specimens from blood samples of OSCC patients (*n* = 47) receiving Cisplatin and 5-Fluorouraccil followed by 40 Gy at 2 Gy per fraction radiation dosage [54]. Analysis showed high pre-treatment serum miR-193b levels predicted pCR (*p* < 0.001), defined as patients with 0% viable tumour cells at the post-treatment stage. AUROC analysis showed strong predictive power in differentiating between responders (*n* = 24) and non-responders (*n* = 23) (AUROC = 0.89, 95% CI: 0.79–1.00, *p* < 0.001), although this was not assessed on any external data. Findings from a study by Wen et al. (2016) comprised of 106 OSCC patients [53], supported observations by Chan et al. (2017), showing that miR-193-3p was downregulated in non-responders (*n* = 17) versus responders (*n* = 89), whereas miR-152, 145-5p and 376a-3p were upregulated in non-responders. An SVM–RBF (Support Vector Machine with Radial Kernel Function) model showed excellent discrimination in distinguishing responders from non-responders using pre-treatment expression of serum miRNAs, with an AUROC of 0.86 (95% CI 0.77–0.97).

#### 3.2.2. Articles Studying Response to nChemo in OSCC Patients

Conversely to the results of both Chan et al. (2017) [54] and Wen et al. (2016) [53], Niwa et al. (2019) [58] suggested that pre-treatment serum expression of miR-193b-5p (*p =* 0.004) and miR-873-3p (*p =* 0.001) were both significantly higher in non-responders (*n* = 69) compared to responders (*n* = 15). AUROC values for Niwa et al. (2019) Logistic regression models were here less discriminative with AUROCs of 0.61 (95% CI 0.47–0.74) for miR-193b-5p and 0.68 (95% CI 0.54–0.81) for miR-873-3p respectively, representing fair performance only and no validation on cases not used to derive the models [58].

Komatsu et al. (2016) [60] and Kurashige et al. (2012) [57] both produced results related to miR-21 that supported its predictive value. Komatsu et al. (2016) [60] took pre-treatment plasma and tissue samples (*n* = 37) from patients undergoing Cisplatin and 5-fluorouracil chemotherapy only. Low histopathological response was classified as JES grade 0 or 1a. MiR-21 expression was found to be significantly higher (*p =* 0.0416) in patients with poor histopathological response (*n* = 17), vs. responders (*n* = 13), with fair ability to discriminate cases (AUROC 0.68). Kurashige et al. (2012) showed that in 71 OSCC patients a significant decrease in miR-21 expression during therapy indicates a higher likelihood of pathological complete response or partial response (*p* = 0.003) [57]. Response was defined according to RECIST 1.0 criteria where pathological complete response is defined as complete disappearance of all tumour lesions on post NAT (pre-surgery) imaging. Thus, the results of both studies were in concordance and suggestive that low pre-treatment concentrations of miR-21 are predictive of a good response to NAT [57,60].

Tanaka et al. (2013) studied pre-treatment serum samples from OSCC patients (*n* = 64) receiving either Adramycin, Cisplatin and 5-Fluorouracil (ACF) or Docetaxel, Cisplatin and 5-Fluorouracil (DCF) [59]. Serum concentrations of miR-200c was only found to be significantly lower in responders (*n* = 39) compared to non-responders (*n* = 25) for the ACF treatment group (*p =* 0.012) versus DCF (*p =* 0.7167). Complete response was defined as total regression of the tumour.

Finally, Komatsu et al. (2016) found pre-treatment plasma concentration of miR-23a were significantly higher in patients with a low histopathological response (*n* = 24, *p =* 0.0345) versus a high histopathological response (*n* = 13 *p =* 0.0125) [56]. AUROC analysis showed relatively strong differentiation between high and low histopathological response groups with an AUROC of 0.70. Logistic regression analysis also revealed that high pre-treatment miR-23a concentrations proved to be an independent risk factor for chemoresistance (*p* = 0.0213; OR: 12.4; 95% CI 1.45–105.8).

### 3.3. MiRNAs Used to Identify Response in Both OAC and OSCC Patients

Two studies looked at both OAC and OSCC histological subtypes (Table 3), both utilised FFPE samples. One articled studied patients treated with nChemo only while the other studied patients treated with nCRT.

#### 3.3.1. Articles Studying Response to nChemo in Both OAC and OSCC Patients

Ko et al. (2012) (*n* = 25; 80% OAC, 20% OSCC) took pre-treatment specimens from patients receiving Cisplatin and Irinotecan followed by 50.4 Gy total dose radiotherapy [62]. Levels of miR-296 were 2.5 times lower (*p =* 0.007) and miR-141 was 2 times higher (*p =* 0.019) in pre-treatment specimens of patients achieving pathological complete response (*n* = 8). Complete response was defined as 0% viable tumour cells remaining. No logistic regression or AUROC was performed to test accuracy of prediction.

#### 3.3.2. Article Studying Response to nCRT in Both OAC and OSCC Patients

Odenthal et al. (2013) [63] (*n* = 80; 52.5% OAC, 47.5% OSCC) used pre-treatment biopsies and showed pre-therapeutic intratumoral expression of miR-192 and miR-194 were significantly higher in major responders (*n* = 11) with OSCC only (*p =* 0.005, *p =* 0.001), classified as Cologne Regression Grade (CRG) III (near complete regression with <10% VRTC) or Grade IV (complete regression/pathologic complete response) [63].

### 3.4. Evidence from In Vitro Functional Studies and Animal Models to Investigate the Utility of miRNAs in Predicting Response to NAT in OAC and OSCC

There is minimal research in the areas of in vitro functional genomics and animal models pertaining specifically to the use of miRNAs in the prediction of oesophageal cancer treatment. However, the few studies that are available have shown promising results which often reproduce results seen in vivo from previously discussed studies in this review (see Table 1, Table 2 and Table 3), particularly relating to miR-193b, miR-21 and miR-200c.

Hummel et al. (2014) investigated miRNA expression in 5-Fluorouracil and Cisplatin chemotherapy resistant cell lines for OAC and OSCC compared to their chemotherapy sensitive variants (OE19 and KYSE410) [64]. MiR-193b-3p was found to be most significantly upregulated in 5-Fluorouracil resistant OAC cell lines with a 1.54-fold increase when compared to controls (*p* ≤ 0.05). These changes were consistent with negative post-transcriptional control of *KRAS* gene expression with its associated mRNAs having a 1.24-fold reduction in expression (*p* = 0.036). The activity of this miRNA in post-transcriptional control with a direct impact on chemosensitivity of the cell, suggests miRNAs do play a mediatory role in chemotherapy resistance. These results support the human data produced by Niwa et al. (2019) [58] and conflict the evidence produced by Chan et al. (2017) [54] and Wen et al. (2016) [53]. However, both Hummel et al. (2014) [64] and Niwa et al. (2019) [58] utilised nChemo only, whereas both Chan et al. (2017) [54] and Wen et al. (2016) [53] utilised nCRT. Thus, the upregulation of miR-21 in neoadjuvant chemotherapy is associated with non-response whereas its upregulation in neoadjuvant chemoradiotherapy was associated with complete pathological response [53,54,58,64].

Hiyoshi et al. (2009) took 20 matched normal oesophageal epithelial samples and oesophageal OSCC samples, as well as seven OSCC cell lines (TE6, TE8, TE10, TE11, TE12, TE14 and KYSE30) to evaluate the role of miR-21 and the effect of anti-miR-21 transfected cell lines [65]. Results showed 18 of the 20 matched OSCC samples overexpressed miR-21 versus normal epithelium (*p* < 0.001). All seven of the cell lines transfected with anti-miR-21 showed a significant reduction in cellular proliferation and invasion, measured via TaqMan real-time PCR of cell lines T6 (1.8-fold reduction), TE8 (1.25-fold reduction) and TE10 (5-fold reduction).

Mahawongkajit et al. (2020) examined two sets of cultured 5-FU resistant OSCC cell lines, TE10-5-FUR and TE11-5-FUR [66]. Each cell line was then compared to its control parent cell line (5-FU sensitive) via a miRNA microarray to determine differences in miRNA expression profiles. Results showed sets of miRNAs displaying the same responses in both cell lines. MiR-146a and miR-483-5p were both significantly upregulated in TE10-5-FUR and TE11-5-FUR cell lines with a 5.59- and 6.19-fold increase, respectively, versus parent control cell lines. For the same cell lines, most significantly in contradiction with Tanaka et al. (2013) [59], miR-200c was collectively downregulated in both 5-FU resistant cell lines by a factor of 4.10. This may be explained by the different types of chemotherapy agents used between the two studies. Mahawongkajit et al. (2020) used cell lines resistant to 5-FU only and thus 5-FU as its only chemotherapeutic agent [66], in contrast to Tanaka et al. (2013) [59] who administered a combination therapy of either ACF or DCF [59]. 5-FU is a potent inhibitor of thymidylate synthetase (TS), which is a key player during thymidylate biosynthesis and hence an essential precursor for DNA synthesis [67]. miR-200c has also be shown to cause inhibition of the same enzyme [68]. Therefore, when 5-FU is used in isolation downregulation of miR-200c would be expected due to the lack of active TS to be inhibited. Thus, it could be concluded that downregulation of miR-200c predicts complete response in neoadjuvant combination chemotherapy via drugs such as Adriamycin, cisplatin and docetaxel used in tandem with 5-FU, as originally suggested by Tanaka et al. (2013) [59].

## 4. Discussion

**Figure 4:**
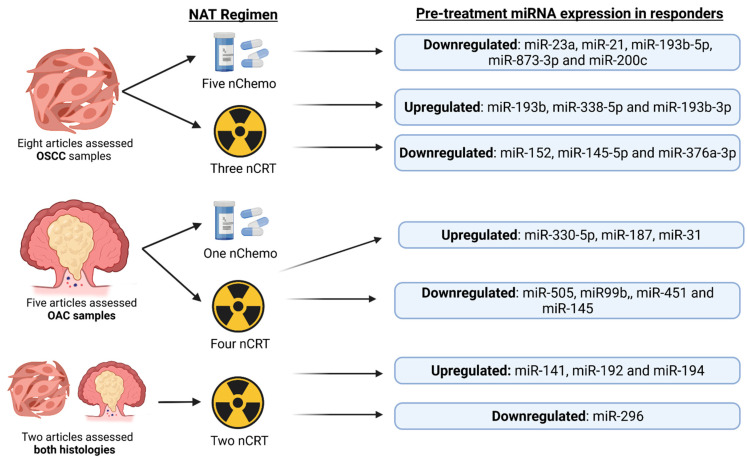
Overview of results produced by all 15 discussed articles. The NAT regimen utilised and how this relates to pre-treatment expression profiles in responders to nChemo or nCRT is shown. Abbreviations: OSCC; Oesophageal Squamous Cell Carcinoma, OAC; Oesophageal Adenocarcinoma, nChemo; Neopadjuvant chemotherapy, nCRT; Neoadjuvant Chemoradiotherapy. (Created with BioRender.com, accessed on 17 February 2022).

The ability to predict OC in patients that will respond to NAT is vital to improve clinical outcomes whilst reducing treatment associated morbidity. This review demonstrates the potential utility of miRNAs as biomarkers of response (Figure 4), with miR-193b, miR-21 and miR-200c showing the most promising results. However, the utilization of singular miRNAs to predict response to NAT is unlikely to be as sensitive or specific as looking at the miRNA expression profiles of multiple miRNAs. The findings of Wen et al. (2016) [53] and the utilization of machine learning models such as SVM–RBF are likely to prove most beneficial, with the latest research showing excellent ability for machine learning techniques to predict events such as recurrence of OC after surgery [69] when looking at postoperative histopathological characteristics. This same model could be utilized preoperatively, looking at miRNA expression profiles to discern whether patients are likely to respond to NAT.

In clinical practice, there are currently no means of stratifying patients into responders and non-responders to NAT in OC. The implementation of miRNA screening prior to the initiation of NAT would allow for patients and the healthcare professionals supporting them to make a more informed decision as to whether treatment is likely to prove beneficial. This is important due to the risks of NAT and the effect it has on the limited length of time and quality of life patients may have. By utilizing a method of predicting response to NAT, such as miRNA screening, in conjunction with new non-invasive diagnostics such as Cytosponge™ technology [25,70] a minimally invasive widespread screening programmed in those at high risk of OC could be formulated. However, research would be needed to understand the ability to predict response utilizing miRNAs from pretherapeutic Cytosponge™ samples.

Despite the number of biomarkers discovered and studied, less than 0.1% are utilized in clinical practice [71]. Most often, this is a result of restricted study design and insufficient sample size or representation. In practice, utilization of miRNAs could surpass these clinical hurdles via the use of multiple different models for miRNA expression in patients with OAC versus OSCC and those receiving nChemo versus nCRT. Establishing the clear differences in miRNA expression between treatment types and doses, and linking these with OC histology, is a key step in establishing miRNAs as clinically viable biomarkers. For each set of treatments, a well-designed study with a large sample size and accurate measurements of predictability, such as AUROC, would prove robust enough for potential implementation into practice. Despite this, there are considerable obstacles for the application of pre-treatment miRNA testing within clinical practice. For example, standardisation in the extraction of miRNAs from either tissues or body fluids. Studies have shown miRNA concentrations in samples can not only differ between tissues and bodily fluids but also be directly affected by the method of extraction itself [72,73]. Further to this, when assessing circulating fluid samples miRNA concentration differs between subfractions (e.g., whole blood, peripheral blood mononuclear cells and plasma), thus it is important to standardise the method of extraction and the subfraction from which miRNAs are to be studied [74]. Studies identifying circulating miRNAs that can predict response to NAT are much more likely to be valuable in clinical practice due their greater accessibility and obtaining these samples is less invasive compared to tissue biopsies. Articles reviewing intratumoral miRNA concentrations are more useful in determining the functional mechanisms by which miRNA expression links to how patients respond to nChemo/nCRT. In addition to this, assessing miRNA expression in vitro often leads to differential results between samples due to the interplay between miRNA expression and the intratumoural microenvironment [49,75]. Based on this, future translational research must focus on the standardisation of miRNA sampling and extraction in circulating fluids, in order to become robust enough biomarkers to use in clinical practice.

### 4.1. Current Use of miR-21, miR-193b and miR-200c in Cancers and Their Functional Roles

This review has provided evidence suggesting that miRNAs may be robust biomarkers for predicting response to NAT by differentiating between responders and non-responders. These studies suggested miR-505, miR-99b, miR-45 (for OAC patients) and miR-193b (for OSCC patients) are accurate biomarkers for predicting response to NAT. In the literature presented in this review the three miRNAs that have consistently appeared significant in both histological OC subtypes, namely OAC and OSCC, are miR-21, miR-193b and miR-200c thus their overall function in OAC and OSCC and other common cancers is discussed.

MiR-21 is a commonly dysregulated in a wide variety of cancers such as renal carcinoma, non-small cell lung cancer, gastric cancer, colon cancer and breast cancer [76]. In oesophageal cancer, high miR-21 has been associated with increased stromal fibroblast activity and increased cell migration [77]; therefore, it is thought to act as an oncogene during the neoplastic life cycle of OSCC with its function being less clear in OAC [76,78]. Studies suggest miR-21 is a useful biomarker in the prediction of response to other cancers such as *HER2* positive breast cancer and colorectal cancers [79,80].

The absolute role of miR-193b in oesophageal cancer is not fully understood, despite the miRNA being known to act as a TSG in various types of gastric and colon cancer [81,82]. Various studies have shown miR-193b initiates apoptosis via the Akt pathway such as in gastric cancers or promotes autophagy and non-apoptotic cell death thereby sensitising cells to chemotherapy [82]. In oesophageal cancer, miR-193b directly targets the *KRAS* pathway and thus, as discussed previously for Hummel et al. (2014), its upregulation in the state of cancer would be expected as it exerts negative transcriptional control to halt cellular proliferation [64]. A 2013 study suggested that dosages of ionizing radiation can manipulate the expression profile for miR-193b in some cancers [83], as supported by the results of Chan et al. (2017) [54] and Wen et al. (2016) [53] whereby miR-193b’s expression profile differed with the addition of radiotherapy. Therefore, this could have affected the patient’s expression profiles should their radiation dosages have differed between patients.

Despite the conclusions made by Tanaka et al. (2013) [59] and Mahawongkajit et al. (2020) [66] there is little conclusive evidence as to the exact function of miR-200c. The miR-200 family have been shown to be tumour suppressor genes in ovarian cancers [84] in addition to their downregulation upon neoplastic progression of Barrett’s oesophagus [85]. There is additional evidence that miR-200c overexpression may play a role in chemoresistance of oesophageal cancers via also interacting with the Akt pathway [86].

### 4.2. Strengths and Limitations of the Included Articles

Of the 15 articles identified 14 suggested that at least one or more miRNAs could be used to predict response to NAT. In total, 10 of the 15 included studies utilised a pre-determined validated measures of analysing response to treatment therapy, these being the defined tumour regression scales of Mandards, Japan Esophageal Society, Cologne Regression Grade and RECIST 1.0 criteria. The other seven studies used generic measures of response (i.e., Tanaka et al. (2013) [59] utilised “total regression of tumour”). The use of generic means of measuring response reduces the ability to draw comparisons between studies, not only making appraisal more difficult, but reducing validity via decreasing repeatability.

Ilhan-Mutlu et al. (2015) [52] is the only study to find no predictive value in any of the miRNAs it analysed, namely miRNA-21 and miR-148a. This is despite the findings previously mentioned by Kurashige et al. (2012) [57] and Komatsu et al. (2016) [60] who found miR-21 was predictive of low response to nChemo in OAC. However, notably with Ilhan-Mutlu et al. (2015) [52] samples used were of SCC histology, which may explain the differing findings. Unlike Ilhan-Mutlu et al. (2015) [52], these studies utilised AUROC to validate the accuracy of predicting and differentiating between responders and non-responders to NAT, further validating their findings and improving external validity. Selection bias was an issue in many of the studies with the most common problems being that of gender bias, small sample sizes or not providing pre-treatment cancer stages for patients. For example, Ilhan-Mutlu et al. (2015) [52] did not provide pre-treatment TNM stages of patients. Thus, concluding whether a lack of response is caused by a cohort of patients entering at a very late stage of presentation (although clinical staging correlates poorly with response) or simply an innate non-response due to other genetic factors, is not possible. Further to this, the breakdown for the number of patients assigned to each chemotherapy regimen does not add up to the total number of patients stated to have taken part in the study (34 in breakdown vs. 36 total participants). Inconsistencies such as these coupled with a relatively narrow age range and small cohort of only one patient responder, reduce validity and likely account for its lack of positive findings. Samples from Kurashige et al. (2012) [57] may have also developed representivity issues because of cohort attrition caused by a fall in participant numbers from 71 to 24. The data set provided on cohort characteristics accounts for the original 71 participants only, therefore it is unknown whether the cohort of patients left over is representative of the general population. However, this was a reasonable change as only 24 serums samples could be obtained both pre- and post-chemotherapy and thus only these samples could be analysed for intra-treatment reductions in miR-21.

Although participant characteristics were well classified in Komatsu et al. (2016) [60] they displayed striking similarities to Komatsu et al. (2016) [56], despite not referencing repeated use of cohorts within the methodologies. Not only were the authors almost identical but total number of participants, age ranges and treatment regimens were all identical. The only differences being miR-21 and miR-23a were their studied miRNAs. Repeated use of the same cohorts without expression may be considered poor scientific practice and only reduces how representative data is to the general population. The same issue occurs for Lynam- Lennon et al. (2012) [51] and Lynam–Lennon et al. (2016) [50] as well as Bibby et al. (2015) [49] where participant characteristics match so closely, and the authors carrying out each study, that it is unlikely a different cohort was used. The main issue with these four studies is each reports the most abundant miRNA as being the most predictive; however, utilising the same cohorts should render the same results each time, considering a similar NAT regime was utilised.

Chan et al. (2017) [54] and Wen et al. (2016) [53] concluded that miR-193b downregulation was predictive of non-response, yet Niwa et al. (2019) [56] suggested that its downregulation was predictive of response to NAT. The predictive accuracy of the findings in both Chan et al. (2017) [54] and Wen et al. (2016) [53] were significantly higher than Niwa et al. [58] with AUROC values of 0.89 and 0.86 respectively vs. 0.61. This difference may be due to differences in treatment regimens used. Niwa et al. [58] utilised neoadjuvant chemotherapy only (Cisplatin and 5-Fluorouracil) whereas Chan et al. (2017) [54] and Wen et al. (2016) [53] applied neoadjuvant chemoradiotherapy. This could suggest miR-193b has a role in the regulation of chemosensitivity and radiosensitivity due to its differing expression. Chan et al. (2017) [54] also experienced problems separating healthy biopsy tissue and cancerous tissues. As no standardised measure of response (e.g., Mandards) was used and percentage of viable tumour cells present was the only measure used, the presence of large amounts of healthy tissue within the specimen could have skewed results suggesting patients are responding better than they actually are. Further to this, radiation dosages were not specified. Therefore, it is unknown whether the changes in miRNA expression can be accounted for by the type of therapy or because of their role in predicting response. A 2013 study suggested that dosages of ionizing radiation can manipulate the expression profile for miR-193b in some cancers [83]. Therefore, this could have affected the patient’s expression profiles should their radiation dosages have differed between patients.

## 5. Recommendations for Future Research

Future research should be carried out in areas considering predicting the response to specific forms of neoadjuvant chemotherapy or chemoradiotherapy in pre-treatment samples. Analysing the differences between drug formulations and dosages in terms of their effects on the predictive power of miRNAs is essential in tailoring treatment to patients [87]. Further to this, more research would be required in producing a score-based model/panel which ranks patients based on multiple miRNAs rather than utilising only one or two miRNAs to attempt to predict response. By producing such a model, it may increase the overall predictive accuracy by including a larger breadth of miRNA expression profiles and thereby increasing the robustness of such a model enabling a quicker translation into clinical usage [45,49].

## 6. Conclusions

MiR-21 and miR-200c in OSCC patients could prove to be a useful biomarker for predicting a patient’s response to NAT. MiR-193b shows promising rates of predictability and functional applicability in both OAC and OSCC histologies. These three miRNAs appear consistently significant in terms of response prediction and their role in chemotherapy and chemoradiotherapy resistance. Despite this, research is still needed to elucidate their absolute role in a therapeutic response, and crucially, how the timings of therapeutic administration may affect miRNA expression and thus their value in predicting response. The efforts of future research need to focus on understanding the effects NAT regimes have on the predictive value of each miRNA individually, yet links must be formed to produce a multi-miRNA model for accurate prediction of NAT response with clinical utility to allow optimal patient benefit. Given the low response rate to SOC chemotherapy agents and significantly high mortality and morbidity of OAC and OSCC, robust and predictive biomarkers of NAT response are urgently needed in the clinic and must become a research priority.

## Figures and Tables

**Table 1:** Articles studying the utility of miRNAs in pre-treatment OAC samples to predict response to NAT.

Authors	Sample Size (*N*)	Gender M, F (*N* (%))	Age (Years); Median (Range)	Cancer Type (Storage)	Pre-Treatment Stage	NAT Regimen	miRNA Quantification Method	Key Results of Predictive miRNA Expression in Responders
Bibby et al. (2015) [49]	18	16/18 (89) 2/18 (11)	65 (37–75)	OAC (Frozen)	T*is*–T4 N0–N1 Mx–M0	nCRT: Cisplatin and 5-FU 1.87 Gy/min	qPCR	↑ miR-330-5p miR-330-5p downregulated in non—responders Responders = Pt with TRG 1-2
Ilhan–mutlu et al. (2015) [52]	36	32/36 (89) 4/26 (11)	* 62 ± 9	OAC (FFPE)	Not specified	nChemo only: Docetaxel (*n* = 8) Mitomycin/Cisplatin/5-FU (*n* = 8) Cisplatin/5-FU (*n* = 10) Epirubicin/Oxaliplatin/Capecitabine (*n* = 1) Others (*n* = 6)	RT-qPCR	No predictive value miR-21 and miR-148a not associated with response prediction pCR = Mandards TRG 1
Lynam–lennon et al. (2016) [50]	18	16/18 (89) 2/18 (11)	* 63 (37–75)	OAC (Frozen)	Stage IIa, IIb and III	nCRT: Cisplatin and 5-FU 40.05 Gy in 15 daily fractions	TaqMan miRNA assay RT-PCR	↑ miR-187 miR-187 expression significantly decreased in pre-treatment biopsies in responders Responders = Pt with TRG 1-2
Lynam–lennon et al. (2012) [51]	19	17/19 (89) 2/19 (11)	* 63 (37–75)	OAC (Frozen)	Stage IIa, IIb and III	nCRT: Cisplatin and 5-FU 40.05 Gy in 15 daily fractions	TaqMan miRNA assay qPCR	↑ miR-31 miR-31 expression is significantly higher in good responders. ‘Good’ responders = Pt with TRG 1-2
Skinner et al. (2014) [45]	Discovery cohort 10 Model Cohort 43 Validation Cohort 65	Discovery cohort 9/10 (90) 1/10 (10) Model Cohort 40/43 (93) 3/43 (7) Validation Cohort M = 63/65 (97) F = 2/65 (3)	Not specified	OAC	Stage II, III and IV	nCRT: Cisplatin and 5-FU 50.4 Gy total dose	Taqman array Fluidigm array Illumina array	↓ miR-505 ↓ miR-99b ↓ miR-451 ↓ miR-145 miR-505/99b/451/145 all showed reduced expression in tumour specimens patients achieving pCR pCR = Complete absence of tumour cells in resected specimen

Abbreviations: * = Mean age, ↑ = Increased expression, ↓ = Decreased expression, Formalin Fixed -Paraffin Embedded (FFPE), Neoadjuvant Chemoradiotherapy (nCRT), Neoadjuvant Chemotherapy (nChemo), Oesophageal Adenocarcinoma (OAC), Oesophageal Squamous Cell Carcinoma (OSCC), Pathological Complete Response (pCR), Reverse Transcription–Quantitative Polymerase Chain Reaction (RT–qPCR) and Tumour in situ (Tis), Tumour Regression Grade (TRG).

**Table 2:** Articles studying the utility of miRNAs in pre-treatment OSCC samples to predict response to NAT.

Authors	Sample Size (*N*)	Gender M, F (*N* (%))	Age (Years); Median (Range)	Cancer Type (Storage)	Pre-Treatment Stage	NAT Regimen	miRNA Quantification Method	Key Results of Predictive miRNAs in Responders
Chan C et al. (2017) [54]	47	44/47 (94) 3/47 (6)	44–82	OSCC	T1–4 N0–N1 M0–M1	nCRT Cisplatin and 5-FU 40 Gy at 2 Gy per fraction	TaqMan Low Density Array RT-PCR	↑ miR-193b High pre-treatment serum miR-193b associated with pCR pCR = Pt with 0% viable tumour cells.
Han et al. (2019) [55]	104	93/104 (89) 11/104 (11)	21 Pt ≤ 55 yrs 83 Pt > 55 yrs	OSCC	T1–4 N0–N3 M0–M1	nCRT Cisplatin and 5-FU [Radiation dose not specified]	RT-qPCR	↑ miR-338-5p High pre-treatment serum miR-338-5p predicts pCR pCR = Pt with JES Grade 1 (ypT0N0M0)
Komatsu et al. (2016) [56]	37	30/37 (81) 7/37 (19)	19 Pt < 65 yrs 18 Pt ≥ 65 yrs	OSCC	Stage I, II, III and IV	nChemo only Cisplatin and 5-FU	Toray^®^ 3D-Gene miRNA array RT-PCR	↓ miR-23a Pre-treatment plasma concentrations of miR-21 were significantly higher in Pt with a low pathological response. Low histopathological response = Pt with JES system TRG 0-1a
Komatsu et al. (2016) [60]	37	30/37 (93) 7/37 (19)	19 Pt < 65 yrs 18 Pt ≥ 65 yrs	OSCC	cT0-4 cN0-3 CM0-1	nChemo only Cisplatin and 5-FU	RT-PCR	↑ miR-21 Expression significantly higher in Pt with low histopathological response Low response = JES TRG 0-1a
Kurashige et al. (2012) [57]	71	66/71 (93) 5/71 (7)	35 Pt < 70 yrs 36 Pt ≥ 70 yrs	OSCC	Stage I, II, III and IV	nChemo only Docetaxel/Cisplatin/ 5-FU (DCF) (*n* = 38) Others (*n* = 33)	TaqMan microRNAs assay qRT-PCR	↓ miR-21 Significant decrease in serum miR-21 was noted in the responders during nChemo. pCR = According to RECIST 1.0 criteria, disappearance of all tumour lesions
Niwa et al. (2019) [58]	84	70/84 (83) 14/84 (17)	65 (30–77)	OSCC	pT0-4 pN0–3 pM0-1	nChemo only Cisplatin plus 5FU or S-1.	Toray 3D-Gene miRNA array RT-PCR	↓ miR-193b-5p ↓ miR-873-3p Pre-treatment serum expression of miR-193b-5p and miR-873-3p were significantly higher in non-responders Responders = Pt with JES Grade 2-3
Tanaka et al. (2013) [59]	64	M = 49/64 (77) F = 15/64 (23)	(67.5) 45–80	OSCC	Stage II, III and IV	nChemo only Adriamycin, Cisplatin and 5-fluorouracil (ACF) OR Docetaxel, Cisplatin and 5-fluorouracil (DCF)	TaqMan Array qPCR	↓ miR-200c Pre-treatment serum levels of miR-200c were significantly lower in responders pCR = Total regression of the tumour
Wen et al. (2016) [53]	106	M = 90/106 (85) F = 16/106 (15)	55 49 Pt < 55 yrs; 57 Pt ≥ 55 yrs	OSCC (Frozen and FFPE)	Stage IIB and III	nCRT only	qPCR	↑ miR-193b-3p ↓ miR-152 ↓ miR-145-5p ↓ miR-376a-3p miR-193-3p was downregulated in pre-treatment biopsies of non-responders. miR-152/145-5p/376a-3p were upregulated in non-responders pCR = 0% residual cancer

Abbreviations: ↑ = Increased expression, ↓ = Decreased expression, Formalin Fixed-Paraffin Embedded (FFPE), Japanese Esophageal Society (JES), Neoadjuvant Chemoradiotherapy (nCRT), Neoadjuvant Chemotherapy (nChemo), Oesophageal Adenocarcinoma (OAC), Oesophageal Squamous Cell Carcinoma (OSCC), Pathological Complete Response (pCR), Response Evaluation Criteria in Solid Tumours (RECIST), Reverse Transcription–Quantitative Polymerase Chain Reaction (RT–qPCR) and Tumour in situ (Tis), Tumour Regression Grade (TRG).

**Table 3:** Articles studying the utility of miRNAs in pre-treatment OAC and OSCC samples combined to predict response to NAT.

Authors	Sample Size (*N*)	Gender M, F (*N* (%))	Age (Years); Median	Cancer Type (Storage)	Pre-Treatment Stage	NAT Regimen	miRNA Quantification Method	Key Results of Predictive miRNAs in Responders
Ko et al. (2012) [62]	25	Not specified	Not specified	OAC (FFPE) 80% OSCC (FFPE) 20%	T1N1M0 or T2-3N0-1	nCRT Cisplatin and Irinotecan 50.4 Gy	Illumina miRNA BeOAChip microarray	↓ miR-296 ↑ miR-141 Pre-treatment specimen levels of miR-296 were significantly lower in Pt achieving pCR. Pre-treatment levels of miR-141 were upregulated in Pt achieving pCR. pCR = 0% viable tumour cells remaining.
Odenthal et al. (2013) [63]	80	M = 68/80 (85) F = 12/80 (15)	59	OSCC (FFPE) 47.5% OAC (FFPE) 52.5%	Not specified	nCRT Cisplatin and 5-FU 40 Gy dose total	TaqManVR Human microRNAs Array RT–P|CR	↑ miR-192 ↑ miR-194 Pretherapeutic intratumoral expression of miR-192 and miR-194 was higher in major responders. Major response = CRG Grades III and IV

Abbreviations: ↑ = Increased expression, ↓ = Decreased expression, Cologne Regression Grade (CRG), Formalin Fixed -Paraffin Embedded (FFPE), Neoadjuvant Chemoradiotherapy (nCRT), Oesophageal Adenocarcinoma (OAC), Oesophageal Squamous Cell Carcinoma (OSCC), Pathological Complete Response (pCR) and Reverse Transcription–Quantitative Polymerase Chain Reaction (RT–qPCR).
